# Gonadotropin regulation of ankyrin-repeat and SOCS-box protein 9 (ASB9) in ovarian follicles and identification of binding partners

**DOI:** 10.1371/journal.pone.0212571

**Published:** 2019-02-27

**Authors:** Gabriel Benoit, Aly Warma, Jacques G. Lussier, Kalidou Ndiaye

**Affiliations:** Centre de recherche en reproduction et fertilité (CRRF), Département de biomédecine vétérinaire, Faculté de médecine vétérinaire, Université de Montréal, St-Hyacinthe, Québec, Canada; University of Florida, UNITED STATES

## Abstract

Ankyrin-repeat and SOCS-box protein 9 (ASB9) is a member of the large SOCS-box containing proteins family and acts as the specific substrate recognition component of E3 ubiquitin ligases in the process of ubiquitination and proteasomal degradation. We previously identified ASB9 as a differentially expressed gene in granulosa cells (GC) of bovine ovulatory follicles. This study aimed to further investigate ASB9 mRNA and protein regulation, identify binding partners in GC of bovine ovulatory follicles, and study its function. GC were obtained from small follicles (SF: 2–4 mm), dominant follicles at day 5 of the estrous cycle (DF), and ovulatory follicles, 24 hours following hCG injection (OF). Analyses by RT-PCR showed a 104-fold greater expression of *ASB9* in GC of OF than in DF. Steady-state levels of *ASB9* in follicular walls (granulosa and theca cells) analyzed at 0, 6, 12, 18 and 24 hours after hCG injection showed a significant induction of ASB9 expression at 12 and 18 hours, reaching a maximum induction of 10.2-fold at 24 hours post-hCG as compared to 0 hour. These results were confirmed in western blot analysis showing strongest ASB9 protein amounts in OF. Yeast two-hybrid screening of OF-cDNAs library resulted in the identification of 10 potential ASB9 binding partners in GC but no interaction was found between ASB9 and creatine kinase B (CKB) in these GC. Functional studies using CRISPR-Cas9 approach revealed that ASB9 inhibition led to increased GC proliferation and modulation of target genes expression. Overall, these results support a physiologically relevant role of ASB9 in the ovulatory follicle by targeting specific proteins likely for degradation, contributing to reduced GC proliferation, and could be involved in the final GC differentiation into luteal cells.

## Introduction

It is well documented that the cyclic ovarian activity results in profound modifications that require spatio-temporal coordination of proliferation, apoptosis and differentiation of various cell types within the ovarian follicle leading to changes in gene expression [1–4]. During the processes of follicular growth and ovulation, steroidogenic cells including granulosa cells (GC) play a crucial role in the maturation and release of the oocyte. Granulosa cells are a particularly important component of the follicle because they play a critical role in reproductive functions as they contribute to steroid hormone synthesis [1], oocyte maturation [2], and corpus luteum formation after ovulation [4, 5]. The control of GC proliferation and function is complex and depends on the precise regulation and activation of specific target genes. This regulation is essential for normal follicular development and timely production of paracrine factors as it affects the physiological state of the dominant preovulatory follicle. For instance, the transcription of specific genes that control the growth of a bovine dominant follicle is rapidly downregulated or silenced in GC as a result of LH-mediated increases in intracellular signaling [3, 6, 7] while LH upregulates or induces the expression of genes involved in ovulation and luteinization [8]. These observations demonstrate the importance of gene functional studies during the final stages of follicular development and ovulation to better coordinate the ovarian activity. In a previous gene expression profiling study, we identified ankyrin-repeat and SOCS-box protein 9 (ASB9) as a differentially expressed gene in GC of bovine ovulatory follicles following the preovulatory LH-surge [8]. In light of these results, we further investigated the regulation and function of ASB9 in GC of ovarian follicles.

ASB9 is a member of the large SOCS box-containing proteins family. Members of the ASB family have two functional domains, a SOCS box and a variable number of N-terminal ankyrin (ANK) repeats [9]. The SOCS box is a conserved domain present in more than 80 proteins of nine different families [10–12]. Initially described as a suppressor of cytokine signaling, it is now clear that SOCS boxes also play an important role in protein turnover via proteasome-mediated degradation [10, 12]. Within ASB proteins, the SOCS box forms an E3 ubiquitin ligase with elongin B/C, culin 5 and Rbx2 proteins to mediate polyubiquitination and subsequent degradation of specific substrate proteins [12–15]. Ankyrin repeats are motifs stacked side by side to mediate protein-protein interactions [16–18]. Each of the 18 members of the ASB family binds more than one protein but these binding proteins are generally specific to one member of the ASB family [19]. With its SOCS box in C-terminal and its ankyrin-repeats in N-terminal, ASB9 acts as a specific substrate recognition component of E3 ubiquitin ligases and interacts with various proteins including brain type creatine kinase (CKB) [17–20]. ASB9 modulates some of the creatine kinase system activities in cell growth and in the signaling of specific target genes [20, 21].

Some reports have shown that ASB9 is predominantly expressed in the kidney and testes [22], has been linked to colorectal cancer [23] and could also be a biomarker for human breast cancer [24]. However, no information is available about ASB9 precise regulation and function in the ovary. We report here, for the first time, the differential regulation of ASB9 mRNA and protein expression in ovulatory follicles and its induction by hCG/LH prior to ovulation. We also report novel ASB9 binding partners in GC of ovulatory follicles using the yeast two-hybrid approach as well as an insight into ASB9 function in the ovarian follicle.

## Materials and methods

### Cloning and characterization of ASB9

We previously established a cDNA library containing transcripts that are upregulated by hCG in GC of ovulatory follicle (OF) using the suppression subtractive hybridization procedure [8] Following the differential hybridization screening of this library, *ASB9* was identified as a differentially expressed gene in GC of OF as compared to dominant follicles. The full length *ASB9* cDNA (AY438595) and its isoform (AY442176) were characterized following screening of a size-selected GC cDNA library from hCG-induced OF (62).

### Experimental animal model and sample preparations

The regulation of ASB9 expression during follicular development and ovulation was studied using *in vivo* models as previously characterized [3]. Following estrous synchronization with PGF_2α_, normal cycling crossbred heifers were randomly assigned to a dominant follicle group (DF, n = 4), or an ovulatory hCG-induced follicle group (OF, n = 4). In the DF group, the ovary bearing the DF on the morning of day 5 of the estrous cycle (day 0 = day of estrus) was obtained by ovariectomy. The DF was defined as ≥ 8 mm in diameter and growing while subordinate follicles were either static or regressing. The OF were obtained following an injection of 25mg of PGF_2α_ on day 7 to induce luteolysis, thereby promoting the development of the DF of the first follicular wave into a preovulatory follicle. An ovulatory dose of hCG (3000 IU, iv; APL, Ayerst Lab, Montréal, QC) was injected 36 hours after the induction of luteolysis, and ovaries bearing the hCG-induced OF were collected by ovariectomy 24 hours post-hCG. Additional OF were collected at 0, 6, 12, 18, and 24 hours after hCG injection for follicular wall preparation (n = 2 cows/time point). The sample at 0 hour was represented by day 7 dominant follicle. Immediately following ovariectomy, follicles were dissected into preparations of follicular wall (theca layer cells with the attached granulosa cells) [25] or further dissected into separate isolates of granulosa cells [3], and stored at –70°C. Additionally, granulosa cells (GC) were collected from 2 to 4mm small follicles (SF) obtained from slaughterhouse ovaries, and a total of three pools of twenty SF were prepared. Corpora lutea (CL) at day 5 of the estrous cycle were obtained by ovariectomy and were dissected from the ovarian stroma, frozen in liquid nitrogen, and stored at –70°C. All animal procedures were approved by the Animal Ethics Committee of the Faculty of Veterinary Medicine of the University of Montreal.

### mRNA expression analysis

Expression and regulation of *ASB9* mRNA during follicular development and following hCG injection was analyzed by RT-qPCR. Total RNA was extracted from bovine GC collected from follicles at different developmental stages (SF, DF, OF) and CL, and from follicular walls (granulosa and theca layer cells) collected at 0, 6, 12, 18, and 24 hours post-hCG injection. Specific *ASB9* PCR primers (Table 1) were used and *ASB9* mRNA relative expression was calculated using the 2^-ΔΔCt^ method [26] with *GAPDH* as reference gene.

**Table 1:** Primers used in the expression analyses of *Bos taurus* genes by RT-qPCR.

Gene Names		Primer sequence (5'–3')*	Accession no.	AS (bp)
**GAPDH**	Fwd Rv	tgttccagtatgattccacccacg ggttgtctcctgcgacttcaacag	NM_001034034	703
**HIF1A**	Fwd Rv	atgtgaccacgaggaaatgag tagttctcccccggctagtta	NM_174339.3	230
**CKB**	Fwd Rv	tcgccctcggtagagtttatt actcccttagtgggacccttt	NM_001015613	240
**ASB9**	Fwd Rv	tcactgcagatcgtgtgtctc tcttagcagcttcgtggatgg	AY438595	165
**PCNA**	Fwd Rv	aagccactccactgtctccta ttaagtgtgtgctggcatctc	NM_001034494.1	207
**CYP19A1**	Fwd Rv	gaggaggtctgcaatgacttg ggtttgagaaggagagcttgc	NM_174305	167
**CYP11A1**	Fwd Rv	gctggcctatcaccgatatta tgacgaagtcctgagacacgt	NM_176644.2	161

Abbreviations: AS, amplicon size (base pairs); Fwd, forward primer; Rv, reverse primer.

*All primers were designed and validated by the authors. Each primer was used at a final concentration of 600 nM.

### Cell extracts and immunoblotting analysis

Granulosa cells and follicular wall preparations obtained as described above, were homogenized in M-PER buffer (Pierce, Rockford, IL, USA) supplemented with complete protease inhibitors (Roche Diagnostics, Laval, QC, Canada), and centrifuged at 16,000 x *g* for 10 min at 4°C. The recovered supernatant was stored at -70°C until electrophoretic analyses were performed. Total protein concentrations were determined using the Bradford method [27] (Bio-Rad Protein Assay, Bio-Rad Lab, Mississauga, ON, Canada). Immunoblotting experiments were performed as described previously [28]. Samples (25μg of proteins) were resolved by one-dimensional denaturing Novex Tris-glycine gels (Invitrogen, Burlington, ON, Canada) and transferred onto polyvinylidene difluoride membranes (PVDF; GE Healthcare Life Sciences). Membranes were incubated with specific first antibodies against ASB9 (rabbit polyclonal anti-ASB9, cat.# ab97918, Abcam; at 5ng/μl final concentration). The immunoreactive proteins were visualized by incubation with appropriate horseradish peroxidase-linked secondary antibodies with the enhanced chemiluminescence system, ECL plus (GE Healthcare Life Sciences), according to the manufacturer’s protocol, and followed by revelation using the ChemiDoc XRS+ system (Bio-Rad).

### Yeast two-hybrid assay

#### Material and media legend

The material and media used for the yeast two-hybrid assay have been previously reported [7].

#### ASB9 constructs for bait preparation

An *ASB9* construct was generated by PCR amplification of a 1593 bp-fragment corresponding to the full-length bovine *ASB9* (AY438595). The PCR product was purified and cloned in frame to the GAL4-DNA binding domain into the pGBKT7 vector to produce a bait plasmid using the Matchmaker Gold Yeast Two-Hybrid System (Clontech) as previously described [7]. The bait plasmid (pGBKT7-ASB9) was used to transform Y2HGold yeast strains and referred to as Y2HGold[pGBKT7-ASB9]. Y2HGold yeast cells harbor four reporter genes (*HIS3*, *ADE2*, *MEL1* and *AUR1*) under the control of *GAL4* upstream activating sequences, which are used to detect two-hybrid interactions. Expression of ASB9 protein in yeast cells containing pGBKT7-ASB9 plasmids was verified using anti-ASB9 antibodies in western blot analysis. To confirm that the pGBKT7-ASB9 bait did not autonomously activate the reporter genes in Y2HGold in the absence of a prey protein, competent Y2HGold cells were transformed with pGBKT7-ASB9 and the transformants were plated on appropriated selective agar plates. In parallel, competent Y2HGold cells were transformed separately with pGBKT7-ASB9 and pGBKT7 empty vector (pGBKT7-C) as control to demonstrate that the ASB9 bait protein was not toxic when expressed in yeast cells. All plated yeast cells were incubated at 30°C for 5 days.

#### Generation of GC-cDNA library and construction of the two-hybrid prey library

A bovine GC-cDNA prey library from ovulatory follicles was prepared in Y187 yeast strain using the pGADT7-Rec vector. cDNAs were expressed as fusion to the GAL4 activating domain using the Matchmaker library construction & screening kit (Clontech User manual PT4085-1) as previously described [7]. Total RNA was isolated from GC of ovulatory follicles and used to generate cDNA with Oligo dT (CDSIII) primers. Competent Y187 yeast cells were prepared and co-transformed with pGADT7-Rec plasmid and cDNAs from GC of ovulatory follicles. The transformed yeast cells, referred to as Y187[pGADT7-GC], were plated, collected after a 5-day incubation at 30°C and stored as previously reported [7].

#### Two-hybrid library screening using yeast mating

The screening procedure was performed as previously reported by our laboratory [7]. Briefly, Y2HGold yeast cells carrying the bait plasmids (Y2HGold[pGBKT7-ASB9]) were mated with Y187 yeast harboring the bovine GC-cDNA library (Y187[pGADT7-GC]). Target prey plasmids responsible for the activation of reporter genes were rescued, isolated and characterized by sequencing. Nucleic acid sequences were verified for the presence of an open reading frame fused in frame to the *GAL4* AD sequence and were compared to those in GenBank.

#### Co-IP confirmation of protein interactions

Physical interactions between ASB9 and candidate partners were confirmed by *in vitro* co-immunoprecipitation assay using the Matchmaker Co-IP system (Clontech). Plasmid constructs containing ASB9 and potential prey partners were used to co-transfect HEK 293 cells using the CalPhos Mammalian transfection kit (Clontech) as recommended by the manufacturer. The potential prey partners tested were tumor necrosis factor alpha-induced protein 6 (TNFAIP6), hypoxia inducible factor 1, alpha subunit (HIF1A) and cytochrome B (CYTB). These partners were selected based on the number of times they were identified and on their potential roles in the ovarian follicle. Cell lysates were prepared as previously described [28]. Physical interactions between ASB9 and prey proteins were validated and quantified using the ProLabel enzyme complementation assay (Clontech). Luminescent signals were recorded every 5 minutes for 45 minutes using a SpectraMax i3 Multi-Mode microplate reader (Molecular Devices). Relative luminescence units (RLU) were plotted as a function of time in order to quantify the relative importance of protein interactions.

#### Regulation of ASB9 partners during follicular development

The expression of ASB9 partners was analyzed during follicular development by RT-qPCR using total RNA from SF, DF, OF and CL samples described earlier. Relative mRNA expression of *HIF1A* was quantified by RT-qPCR using specific primers (Table 1), and the results were analyzed using the Livak method [26]. Creatine kinase B expression was also analyzed during follicular development and in ovulatory follicles at different hours post-hCG injection.

### Functional studies using CRISPR/Cas9 experiments

To study the function of ASB9 in GC of ovulatory follicles, we used the CRISPR/Cas9 technology through the guide-it CRISPR/Cas9 system (Clontech) for the cloning and expression of target single guide RNAs (sgRNAs) for ASB9 inhibition in GC. Four sgRNAs were designed using online tools to maximize cleavage efficiency at the target site and minimize non-specific cleavage events. The efficiency of designed sgRNAs was tested prior to transfection experiments using the Guide-it sgRNA *in vitro* transcription and Screening System (Clontech). DNA templates containing sgRNA-encoding sequences under the control of T7 promoter were generated by PCR, *in vitro* transcribed, and purified. Cleavage templates for screening the purified sgRNAs were produced by amplification of DNA fragments that contain ASB9 sequence. A cleavage reaction on these templates was performed using the purified sgRNAs with the recombinant Cas9 nuclease (Clontech). The efficiency of cleavage reactions was analyzed on agarose gel followed by densitometry analyses. A sgRNA was identified and used for cloning into the pGuide-it-ZsGreen1 vector for plasmid construct and transfection of GC using the Xfect transfection kit (Clontech). GC were collected from large follicles (≥ 8 mm in diameter) of slaughterhouse ovaries and cultured either in 96-well plates (proliferation assay) or in 24-well plates (gene expression analysis) in DMEM/F12 supplemented with L-glutamine (2 mM), sodium bicarbonate (0.084%), bovine serum albumin (BSA; 0.1%), HEPES (20 mM), sodium selenite (4 ng/ml), transferrin (5 μg/ml), insulin (10 ng/ml), non-essential amino acids (1 mM), penicillin (100 IU) and streptomycin (0.1 mg/ml). Transfected GC along with control GC (transfection with empty vector or no transfection) remained in culture for six days with media replacement every two days. Expression of Cas9 protein in GC was confirmed by western blot analysis with anti-Cas9 antibodies and ASB9 gene editing was confirmed through the presence of mutations using a PCR-based analysis kit (Clontech). The effects of CRISPR/Cas9-induced ASB9 inhibition were assessed by measuring GC proliferation and markers for proliferation, specifically analyzing the expression of PCNA and steroidogenic genes CYP19A1 and CYP11a1. We also analyzed the effects of ASB9 silencing on creatine kinase B (CKB) expression in GC.

### Statistical analyses

Relative amounts of *ASB9* and other target genes mRNA were normalized with those of the control gene *GAPDH*. Homogeneity of variance between groups was verified by O’Brien and Brown-Forsythe tests. Corrected values of gene specific mRNA levels were compared between follicular or CL groups by one-way analysis of variance (ANOVA). When ANOVA indicated a significant difference (P < 0.05), the Tukey-Kramer test was used for multiple comparison of individual means among SF, DF, OF and CL, whereas the Dunnett test (P < 0.05) was used to compare different time points after hCG with 0 hour as control. Data were presented as least-square means ± SEM. Statistical analyses were performed using GraphPad prism 5.0 software.

## Results

### ASB9 expression in granulosa cells is induced by hCG/LH

**Fig 1:**
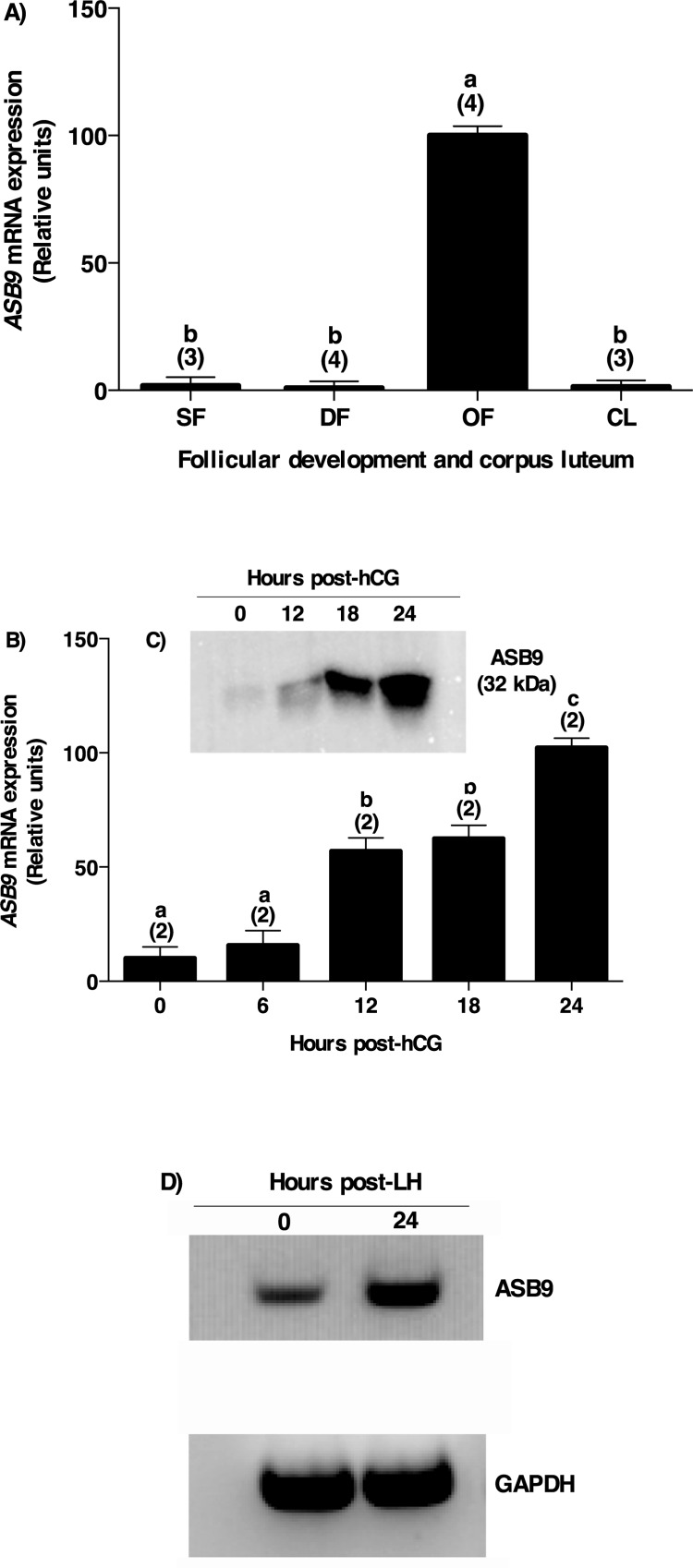
ASB9 expression and regulation in bovine granulosa cells. **A**) Total RNA extracts of bovine granulosa cells from small follicles (SF; 2–4 mm in diameter; n = three pools of 20 follicles), dominant follicles obtained at Day 5 of the estrous cycle (DF; n = 4 animals), ovulatory follicles isolated 24h post-hCG (OF; n = 4 animals), and corpora lutea obtained at Day 5 of the estrous cycle (CL n = 3 animals) were analyzed by RT-qPCR for *ASB9* and *GAPDH* (used as reference gene) mRNA expression. **B**) Similarly, total RNA from bovine follicular walls (theca layer cells with attached granulosa cells) were isolated from OF at 0, 6, 12, 18 and 24 hours (h) post-hCG injection (n = 2 animals per time point) and analyzed by RT-qPCR. *ASB9* relative amounts were normalized with respect to *GAPDH* and presented as least-square means ± SEM. **A)** *ASB9* mRNA expression was increased by 104-fold in OF as compared to DF. **B**) Further analyses showed a significant induction of *ASB9* expression in follicular walls at 12 and 18 h, reaching a maximum induction at 24 h post-hCG injection as compared to 0 h. **C**) Total protein extracts of bovine granulosa cells isolated from OF at 0, 12, 18, and 24 hours post-hCG injection were analyzed by western blot using anti-ASB9 antibodies. hCG induced ASB9 protein expression starting at 12 hours with the strongest expression observed at 24h post-hCG, reflecting the regulation of the mRNA. **D**) Gel analysis of *ASB9* mRNA regulation by endogenous LH. Similar to hCG, induction of *ASB9* expression was observed 24 h after the endogenous LH surge as compared to 0 h. Different letters denote samples that differ significantly (P < 0.05). Bars marked with an asterisk are significantly different from 0 h (P<0.05).

In order to investigate *ASB9* mRNA expression and regulation in bovine follicles, total RNA extracts of bovine GC from small follicles (SF; 2–4 mm in diameter), dominant follicles obtained at day 5 of the estrous cycle (DF), ovulatory follicles isolated 24 hours post-hCG (OF), and corpora lutea obtained at day 5 of the estrous cycle (CL) were analyzed using RT-qPCR. Expression of *ASB9* was significantly increased in ovulatory follicles following hCG injection and reduced to basal levels in SF, DF and CL (Fig 1A; P<0.05). *ASB9* mRNA expression was increased by 104-fold in OF following hCG injection as compared to DF (Fig 1A).

Because *ASB9* transcript was expressed to high amounts in ovulatory follicles, the gonadotropin-dependent regulation of *ASB9* mRNA during the periovulatory period was further investigated in ovulatory follicles using total RNA obtained from follicular walls (theca layer cells with attached granulosa cells) of preovulatory follicles collected at 0, 6, 12, 18 and 24 hours post-hCG injection. The results from RT-qPCR analyses showed a significant induction of *ASB9* expression in follicles at 12 and 18 hours post-hCG (Fig 1B; P<0.05), reaching a maximum induction at 24 hours post-hCG as compared to 0 hour (Fig 1B; P<0.05). This induction of *ASB9* mRNA expression was confirmed with the endogenous luteinizing hormone (LH) surge model showing an upregulation of *ASB9* at 24 hours post LH surge as compared to 0 h (Fig 1D). Furthermore, ASB9 protein expression was investigated by western blot analysis using anti-ASB9 antibodies. Similar to the mRNA expression, results obtained with protein extracts using granulosa cells isolated from ovulatory follicles at 0, 12, 18 and 24 hours after hCG injection showed an induction of ASB9 protein expression at 18 and 24 hours as compared to 0 hour (Fig 1C).

### Yeast two-hybrid (Y2H) screening revealed potential ASB9 partners in granulosa cells

**Fig 2:**
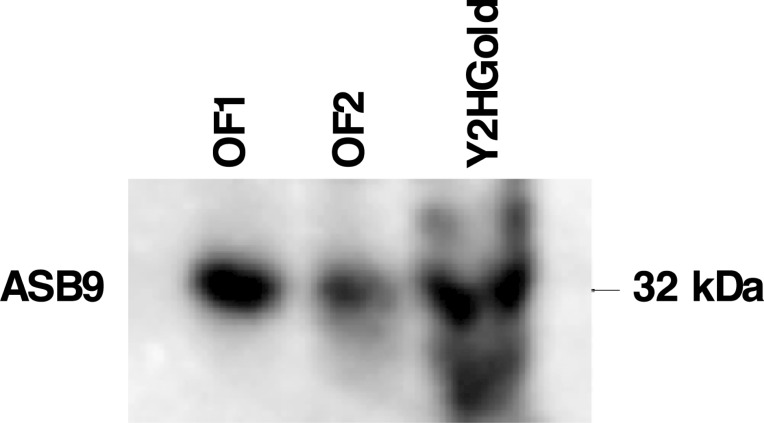
Confirmation of ASB9 expression in yeast cells. Y2HGold yeast strain was transformed with the construct pGBKT7-ASB9. An overnight culture was used to extract protein and perform western blot analysis using anti-ASB9 antibodies. Total protein extracts of bovine granulosa cells from two ovulatory follicles isolated 24h post-hCG (OF1 and OF2) were used as positive controls. Results confirmed ASB9 expression in the Y2HGold yeast strain.

To identify ASB9 binding partners in granulosa cells, a yeast two-hybrid screening was performed. The Y2HGold yeast strain were transformed either with pGBKT7 empty vector as control (pGBKT7-C), or the plasmid construct containing ASB9 (pGBKT7-ASB9), and were spread on selective media to verify for toxicity and autoactivation analyses. The construct pGBKT7-ASB9 was not toxic to the Y2HGold yeast strain and ASB9 did not, by itself, activate the transcription of reporter and selection genes (*AUR-C*, *ADE2*, *HIS3*, and *MEL1*), since no colonies grew when ASB9 construct was plated in the presence of aureobasidin A antibiotic (S1 Fig). To verify that ASB9 protein was expressed in yeast cells, total protein from Y2HGold strain transformed with pGBKT7-ASB9 was used to perform western blot analysis using anti-ASB9 antibodies. Total protein extracts of bovine granulosa cells from two ovulatory follicles isolated 24 hours post-hCG were used as positive controls. Western blot analysis confirmed ASB9 protein expression in the Y2HGold yeast strain (Fig 2).

Finally, mating of the Y2HGold[pGBKT7-ASB9] strain with the Y187[pGADT7-GCcDNA] strain resulted in the presence of zygotes that indicated potential interactions between the bait (ASB9) and prey proteins contained in the ovulatory follicle GC-cDNA library (S2 Fig). A limited number of potentially positive yeast colonies was used to analyze for the presence or absence of a cDNA insert. The presence of a cDNA insert indicates a true positive suggesting a potential partner for ASB9 while the absence of an insert indicates a false positive (S3 Fig). Plasmids from positive colonies were purified from yeast colonies containing an insert, amplified by PCR and sequenced. Sequence analyses showed that yeast two-hybrid screening of ovulatory follicle GC-cDNAs library resulted in the identification of 10 potential ASB9 binding partners in GC, two of which were chosen to be further confirmed (Table 2).

**Table 2:** List of ASB9 binding partners.

Gene Names	Accession #*	Freq.	Ident. (%)	E-value	Description
**CYTB**	JX472273	4	100	0.0	B.T. Cytochrome b
**RGN**	NM_173957	1	99	0.0	B.T. Regucalcin
TNFAIP6**	NM_001007813	1	100	7e-154	B.T. TNF alpha induced protein 6
**PHLDA1**	XM_019960200	1	99	0.0	B.I. Pleckstrin homology like domain family A member 1
HIF1A**	NM_174339	1	93	0.0	B.T. Hypoxia inducible factor 1 subunit alpha
**TUBB4B**	NM_001034663	1	99	8e-78	B. T. Tubulin beta 4B class IVb
**SLC25A15**	NM_001046326	1	95	1e-14	B.T. Solute carrier family 25 member 15
**TAOK1**	XM_024980290	1	99	0.0	B.T. TAO kinase 1
**GLOD4**	XM_019980727	1	88	5e-84	B.I. Glyoxalase domain containing 4
**GATD3A**	NM_001034463	2	99	0.0	B.T. Glutamine amidotransferase like class 1 domain containing 3A

Plasmids were purified from true positive yeast colonies, amplified by PCR and sequenced. Sequences were analyzed for identity and resulted in 10 different proteins potentially interacting with ASB9.

*Accession number of the best match found following nucleotide sequence comparison via BLAST search in GenBank.

Freq.: Frequency of cDNA clone identification from yeast two-hybrid prey library; Ident. (%): Identity: represents homology estimates of bovine prey cDNA fragments with nucleotide sequences in GenBank database; B.T.: *Bos taurus*; B.I.: *Bos indicus*.

**Partners whose physical interactions with ASB9 were confirmed by co-immunoprecipitation assays.

### ASB9 physically interacts with TNFAIP6 and HIF1A

**Fig 3:**
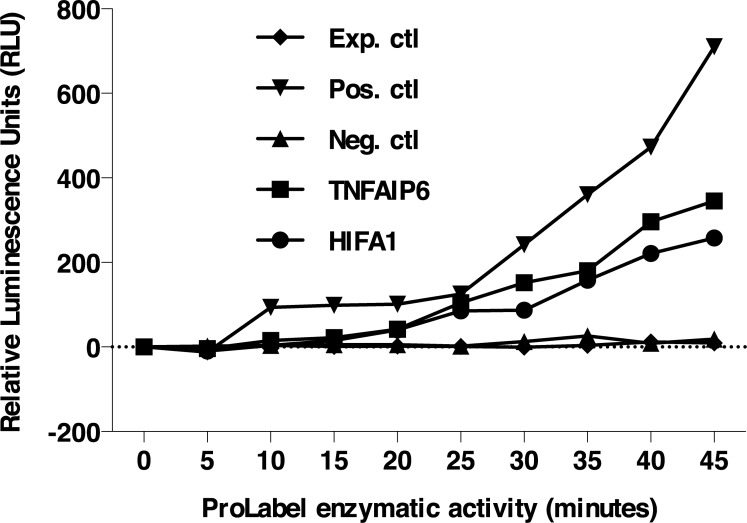
Chemiluminescence analyses and confirmation of ASB9 protein interaction with TNFAIP6 and HIF1A. ASB9 bait was cloned into the pAcGFP1-C vector and the prey partners from OF granulosa cell library (tumor necrosis factor alpha-induced protein 6 [TNFAIP6] and hypoxia inducible factor 1, alpha subunit [HIF1A] were cloned, separately, into the pProLabel-C vector for co-transfection of HEK 293 cells. After co-expression and protein extraction, co-immunoprecipitations using protein G Plus/A agarose beads were followed by chemiluminescence analyses. Using the ProLabel enzyme complementation assay, luminescent signals (expressed in relative luminescent unit [RLU]) of TNFAIP6 and HIF1A were compared to a positive interaction (Pos. ctl) consisting of pAcGFP1-53 and ProLabel-T, to an experimental control (Exp. ctl) consisting of pAcGFP1-ASB9 and ProLabel-empty vector, and a negative control (Neg. ctl). TNFAIP6 and HIF1A RLU were significantly increased as compared to the experimental and negative controls confirming a real physical interaction. After 40 minutes of the substrate addition, there was 18-fold and 13-fold induction in ProLabel enzymatic activity in HEK cells co-transfected, respectively, with ASB9/TNFAIP6 and ASB9/HIF1A as compared to the experimental control.

Included in the group of ASB9 binding partners are tumor necrosis factor alpha-induced protein 6 (TNFAIP6) and hypoxia inducible factor 1, alpha subunit (HIF1A). In order to further confirm yeast two-hybrid results in a mammalian cell model, physical interactions between ASB9 and candidate partners were investigated by *in vitro* co-immunoprecipitation followed by ProLabel enzyme complementation assay in HEK 293 cells. Using the ProLabel enzyme complementation assay, relative chemiluminescent signals (RLU) of TNFAIP6 and HIF1A were compared to a reference positive interaction, an experimental control and a negative control. Similar to the positive control, TNFAIP6 and HIF1A RLU signals were significantly increased as compared to the experimental and negative controls, confirming a physical interaction with ASB9 (Fig 3). After 40 minutes of incubation with the substrate, induction of ProLabel enzymatic activity in HEK cells co-transfected with ASB9/TNFAIP6 or ASB9/HIF1A increased, respectively, by 18- and 13-fold as compared to the negative control (Fig 3). As reference, induction of ProLabel enzymatic activity increased in the positive control by 37-fold.

### ASB9 partners are differentially regulated during follicular development

**Fig 4:**
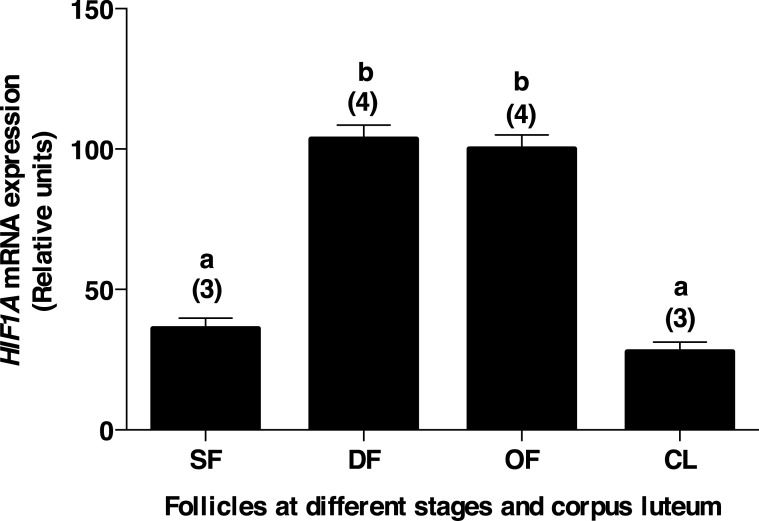
*HIF1A* mRNA expression in bovine granulosa cells. Total RNA extracts of bovine granulosa cells from small follicles (SF n = 3), dominant follicles obtained at Day 5 of the estrous cycle (DF n = 4), ovulatory follicles isolated 24h post-hCG (OF n = 4), and corpora lutea obtained at Day 5 of the estrous cycle (CL n = 3) were analyzed by RT-qPCR for *HIF1A* and *GAPDH* (as reference gene) mRNA expression. *HIFA* relative amounts were normalized with respect to *GAPDH*, and the results are presented as least-square means ± SEM. Steady-state mRNA expression of *HIF1A* was increased by 2.86-fold in DF as compared to SF and remained strong in the OF before declining in the CL. Different letters denote samples that differ significantly (P<0.05).

In order to investigate the regulation of ASB9 binding partners expression during follicular development, total RNA extracts of bovine granulosa cells from small follicles (SF), day 5-dominant follicles (DF), ovulatory follicles isolated 24 hours post-hCG (OF), and day 5-corpora lutea (CL) were analyzed by RT-qPCR for *HIF1A* using specific primers. From SF to DF, *HIF1A* mRNA expression was increased by 2.86-fold (Fig 4; P<0.05) and remained similarly strong in the OF before decreasing significantly in the CL as compared to DF and OF (Fig 4; P<0.05). Significant induction of *TNFAIP6* mRNA expression in OF has already been clearly demonstrated in previous work [29, 30].

**Fig 5:**
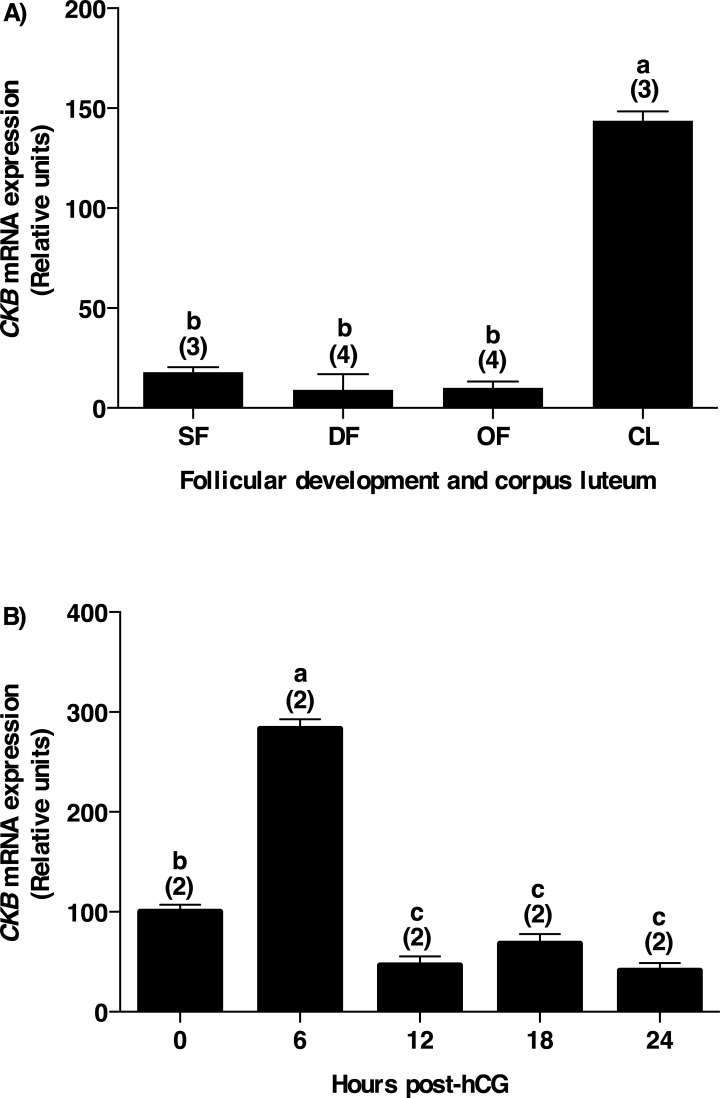
*CKB* mRNA expression in bovine granulosa cells. **A**) Total RNA extracts of bovine granulosa cells from small follicles (SF; n = 3), day 5-dominant follicles (DF; n = 4), 24 hours post-hCG-ovulatory follicles (OF; n = 4), and day 5-corpora lutea (CL n = 3) were analyzed by RT-qPCR for *CKB* and *GAPDH* mRNA expression. *CKB* relative amounts were normalized with respect to *GAPDH* and results are presented as least-square means ± SEM. *CKB* mRNA expression was significantly stronger in the CL as compared to all stages of follicular development. **B**) Total RNA from bovine follicular walls were isolated from OF at 0, 6, 12, 18 and 24 hours post-hCG injection (n = 2 per time point) and analyzed by RT-qPCR. Analyses showed an increase in *CKB* expression in follicular walls at 6h post-hCG as compared to 0h. However, *CKB* expression rapidly declined starting at 12h post-hCG through 24h post-hCG. Different letters denote samples that differ significantly (P<0.05).

Since previous studies have shown interaction between ASB9 and brain type creatine kinase (CKB) [17–20] and no interaction between these two proteins was identified in granulosa cells from our yeast two-hybrid screening, we decided to analyze *CKB* expression during follicular development and verify its expression in granulosa cells as compared to that of *ASB9*. Steady-state mRNA expression of *CKB* was strongest in the CL at day 5 of the estrous cycle as compared to all stages of follicular development (Fig 5A; P<0.05) while *ASB9* expression was strongest in ovulatory follicles (Fig 1A). In addition, *CKB* expression post-hCG injection showed an increase 6 hours post-hCG and a rapid decline at 12 hours post-hCG and remained low through 24 hours post-hCG (Fig 5B). In contrast, *ASB9* expression was strongest 24 hours post-hCG and weakest at 0 and 6 hours post-hCG (Fig 1B).

### Functional studies using CRISPR-Cas9

**Fig 6:**
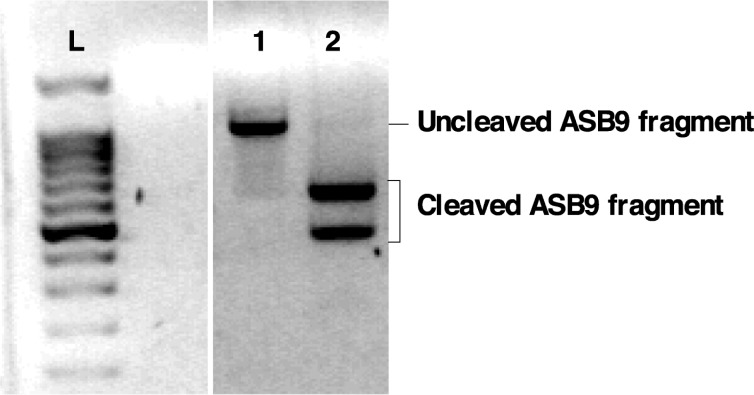
Design and identification of an efficient sgRNA. Four sgRNA sequences were synthesized and tested against ASB9 sequence from GC. The efficiency of cleavage reactions was analyzed on agarose gel and by densitometry. A sgRNA with 97.4% efficiency at directing Cas9-mediated cleavage of ASB9 mRNA was identified. L: 100 bp ladder; lane 1: uncleaved ASB9 fragment; Lane 2: Cleaved fragments. sgRNAs with lower efficiencies are not shown.

Four sgRNA sequences were synthesized and tested against *ASB9* sequence from GC. A sgRNA with 97.4% efficiency at directing Cas9-mediated cleavage of *ASB9* was identified (Fig 6). This sgRNA was used for cloning into the pGuide-it vector for plasmid construct and transfection of bovine GC.

**Fig 7:**
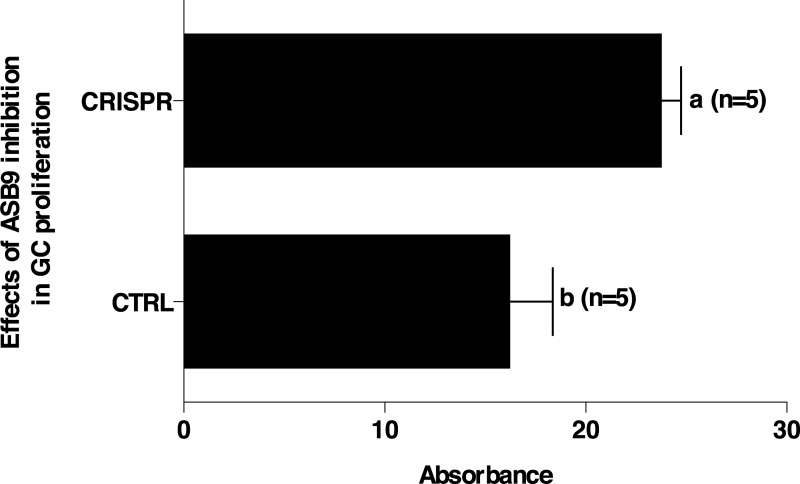
Proliferation assay of GC with CRISPR-Cas9-induced ASB9 inhibition. Bovine GC collected from large follicles were cultured in DMEM/F12 as described in materials and methods. GC were seeded in 96-well plates and incubated at 37°C/5% CO_2_ and proliferation was determined using the CellTiter assay kit (Promega). The CellTiter substrate was added for 3 hours before measuring absorbance at 490 nm with the SpectraMax i3 (Molecular Devices). Inhibition of ASB9 significantly increased GC proliferation (P<0.05).

**Fig 8:**
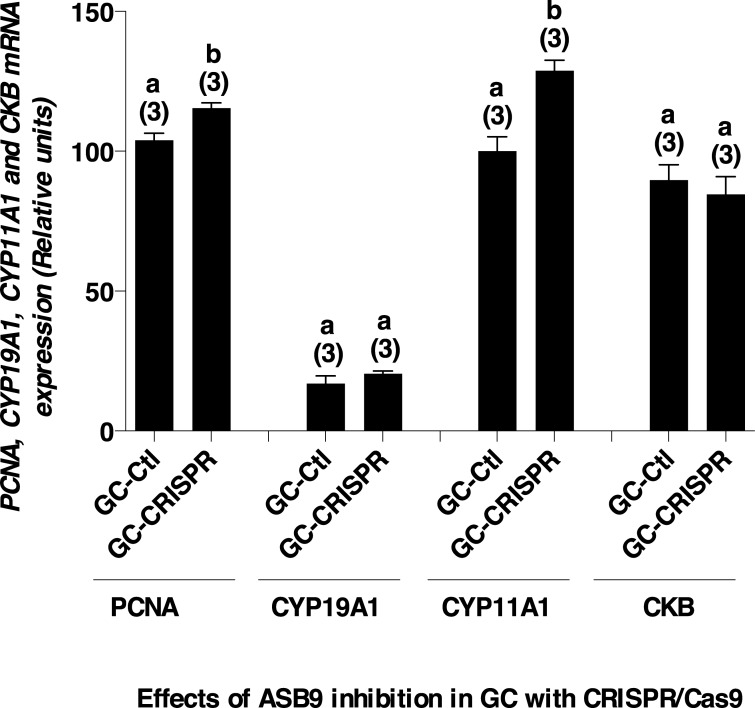
mRNA expression of *PCNA*, *CYP19A1*, *CYP11A1* and *CKB* in ASB9-inhibited GC. Total RNA was extracted from cultured GC following CRISPR-Cas9-induced inhibition of ASB9 and control GC. In addition, total RNA extracts were prepared from bovine granulosa cells of small follicles (SF), dominant follicles (DF), ovulatory follicles (OF), and corpora lutea (CL). Samples were analyzed by RT-qPCR for *PCNA*, *CYP19A1*, *CYP11A1* and *CKB* mRNA expression and relative amounts were normalized with *GAPDH*. *PCNA* expression significantly increased in CRISPR/Cas9-induced inhibition of ASB9 (1.12-fold; Fig 8A). Steroidogenic gene *CYP19A1* expression was not affected by ASB9 inhibition (Fig 8B) while *CYP11A1* expression was significantly increased when ASB9 was inhibited (1.28-fold; Fig 8C). There were no significant changes in *CKB* expression following ASB9 inhibition (Fig 8D). Different letters denote samples that differ significantly (P<0.05).

Effects of ASB9 silencing on cell proliferation and target genes expression were analyzed. Results showed that ASB9 silencing led to significant increase in GC proliferation (Fig 7; P<0.05). In addition, Proliferating cell nuclear antigen (*PCNA*) expression was significantly increased in GC when ASB9 was inhibited using CRISPR/Cas9 (Fig 8A). *PCNA* was used to assess and confirm GC proliferation since it is expressed in the nuclei of cells during the DNA synthesis phase of the cell cycle. To verify the effects of ASB silencing in steroidogenesis, expression of *CYP19A1* and *CYP11A1* were analyzed. Expression of *CYP19A1* expression was not affected by ASB9 inhibition (Fig 8B) while *CYP11A1* expression was significantly increased when ASB9 was inhibited (Fig 8C). Finally, there was no significant changes in CKB expression following ASB9 inhibition (Fig 8D).

## Discussion

During follicular development, granulosa cells along with theca cells and oocyte, exhibit a series of functional changes in terms of gene activation or inhibition. Granulosa cells become more responsive to FSH and show a higher rate of proliferation once a dominant follicle is selected [3, 4, 7], while the preovulatory follicle displays an array of genes induced by the LH surge or hCG injection [8, 29, 31]. In this study, we report for the first time ASB9 regulation and protein interactions in the reproductive system using granulosa cells from bovine ovarian follicles. Our results show that ASB9 expression is hormonally-regulated as it is significantly induced by the endogenous LH surge and by hCG injection in a time-dependent manner. The greatest expression of ASB9 was found in the ovulatory follicle 24 hours post-hCG injection both at the mRNA and protein levels. It is known that ASB9 is involved in the pathway of protein ubiquitination, which is part of protein modification and thus acts as a ubiquitin ligase controlling the abundance of target proteins [18, 20]. Because ASB9 expression in the OF is considerably induced as compared to the growing dominant follicle, it is conceivable that ASB9 might play a role in the ovulatory process and extracellular matrix remodeling by targeting specific proteins for binding and degradation. In fact, in this study, inhibition of ASB9 expression in granulosa cells using CRISPR-Cas9 leads to increased granulosa cell proliferation and modulated specific genes expression including steroidogenic gene CYP11A1. These observations indicate that ASB9 might be associated with controlling the activity of target genes involved in the ovulatory follicle immediately prior to ovulation. These observations could also suggest that ASB9 is involved in granulosa cells differentiation into luteal cells similar to its perceived role in mouse spermatogenesis [22].

Ankyrin repeat and SOCS Box proteins interact with a wide variety of target substrates via ankyrin repeat domains [16–18]. In addition, members of ASB protein family can interact with the elongin B-C adapter complex via their SOCS box domain and further complex with the cullin and ring box proteins to form E3 ubiquitin ligase complexes and participate in protein degradation [10, 20, 32]. Thus, SOCS proteins regulate protein turnover by targeting proteins for polyubiquitination and proteasome-mediated degradation. To our knowledge, no interaction or function of ASB9 has been reported in granulosa cells of any species. Using a yeast two-hybrid screening, we identified and confirmed novel ASB9-interacting proteins in granulosa cells of ovarian follicles that could be targeted by ASB9 in the ovulatory follicle most likely through the pathway of protein ubiquitination. The association between ASB9 and its binding partners could then lead to degradation of target proteins resulting in gene expression changes and possibly contributing to differentiation of steroidogenic cells into luteal cells after ovulation. We identified 10 individual potential partners for ASB9 in bovine granulosa cells, some of which are known to be expressed in ovulatory follicles and participate actively in the ovulation process. Of interest, we showed that TNFAIP6 and HIFA1 interact with ASB9 in granulosa cells.

Tumor necrosis factor alpha-induced protein 6 (TNFAIP6), also known as Tumor necrosis factor-stimulated gene 6 (TSG6), is a secretory protein that contains a hyaluronan-binding domain known to be involved in extracellular matrix (ECM) stability and cell migration [33, 34]. TNFAIP6 has been shown to form a stable complex with inter-α-inhibitor to enhance the serine protease inhibitory activity of inter-α-inhibitor, which is important in the protease network associated with inflammation [35]. TNFAIP6 can also be induced by proinflammatory cytokines such as tumor necrosis factor alpha and interleukin-1 [36, 37]. Moreover, enhanced levels of TNFAIP6 are found in the synovial fluid of patients with osteoarthritis and rheumatoid arthritis [35, 38–40]. It is well established that the ovulation process shares numerous signs of an acute inflammatory reaction [41]. It is therefore consistent that TNFAIP6 expression increases during ovulation process. The preovulatory endogenous LH surge or hCG injection induces the expression of several genes involved in the ovulatory process including TNFAIP6, which is highly induced in follicular cells of rodents and mare [41–45]. Several studies have documented the crucial role of TNFAIP6 in ECM production, cumulus expansion and fertility [42, 46–48]. A Previous study also provided evidence for a marked induction of TNFAIP6 by gonadotropins in follicular cells before the rupture of the follicle in cows [29]. Ovulation also involves a series of events such as ECM formation and cumulus-oocyte complex (COC) expansion leading to the rupture of the preovulatory follicle and release of the oocyte [49, 50]. ASB9 binding to TNFAIP6 could be a regulatory mechanism to facilitate the follicle’s rupture and transition to luteinisation. Although TNFAIP6 could be targeted by ASB9, likely for degradation by the proteasome, the mechanism by which ASB9 binds to TNFAIP6 remains unclear. Furthermore, TNFAIP6 targeting by ASB9 likely occurs right before ovulation, which in the cow occurs around 28 hours post-LH or post-hCG as previously reported [51] and when ASB9 expression is strongest and when TNFAIP6 activity is no longer required for ECM stability or COC expansion. It has been shown in bovine granulosa cells that TNFAIP6 expression dramatically increased at the 6-hour point post-LH and significantly decreased at 22 hours post-LH [52]. Another study, also using bovine granulosa cells, showed a significant induction of TNFAIP6 expression 6 hours post-hCG with the strongest induction at 12 hours post-hCG, although this induction remains strong through 24 hours post-hCG [29]. Both studies suggest a crucial action of TNFAIP6 between 6 and 12 hours post-LH/hCG, suggesting that at 24 hours post-LH/hCG, TNFAIP6 activity could be regulated by ASB9 possibly through ubiquitination and degradation. Induction of various genes by LH/hCG in the preovulatory period varies greatly as shown in bovine and rats [8, 53, 54]. Additionally, ASB9 and TNFAIP6 peak expressions seem to occur at different times after the LH surge or hCG injection suggesting that ASB9 would bind TNFAIP6 only after the latter has participated in critical ovulatory steps.

The second binding partner whose interaction with ASB9 was confirmed is HIF1A. Hypoxia-inducible factor-1 (HIF1) is a heterodimer and a member of the basic-Helix-Loop-Helix-PAS family of transcription factors composed of an alpha and beta subunits [55]. In mammals, HIFs function as regulators of cellular and systemic homeostatic response to hypoxia by activating transcription of many genes whose protein products increase oxygen delivery or facilitate metabolic adaptation to hypoxia [56]. HIF1 activity is controlled by the oxygen-regulated expression of the alpha (HIF1A) subunit. Under normal conditions, HIF1A is subject to ubiquitination and proteasomal degradation but is stabilized by hypoxia [56–58]. Previous studies have shown that HIF1A is activated in FSH-stimulated ovarian cancer cells SKOV-3 [59], as well as in mouse granulosa cells where HIF1A was shown to be an inducible factor after FSH treatment *in vivo* and in *vitro* [60]. In the ovary, excessive cell proliferation induced by gonadotropins promotes the accumulation of HIF1A and leads to hypoxia [61]. In contrast, inhibition of the HIF transcriptional activity suppresses the binding of HIF αβ-heterodimers to target genes and blocks ovulation by preventing the rupture of the preovulatory follicles [62]. In our study, we showed that HIF1A expression in granulosa cells was strongest in the growing dominant follicle and remained strong in the ovulatory follicle before declining in the CL. The strong presence of HIF1A in the dominant follicle is consistent with its accumulation associated with proliferation of granulosa cells in the selected dominant or pre-ovulatory follicle. In contrast, it is possible that the LH surge or hCG injection creates a hypoxic condition within the ovulatory follicle leading to enhanced HIF1A expression during ovulation. Increased expression of HIF1A during the ovulatory period may occur due to enhanced hypoxia-induced stability of HIF1A as suggested previously [62]. However, ASB9 would eventually target HIF1A for degradation as steroidogenic cells are transitioning from a proliferative state to a final state of differentiation into luteal cells.

ASB9 has been shown to bind to and ubiquitinate brain-type cytosolic creatine kinase (CKB) [18, 20, 63] and ubiquitous mitochondrial creatine kinase (uMtCK) [32, 64]. Interaction between ASB9 and CKB resulted in the SOCS box-dependent ubiquitination and proteasomal degradation of CKB while ASB9 overexpression dramatically reduced endogenous CKB protein [20] and negatively regulates cell growth [32]. In the present study, interaction between ASB9 and CKB was not found in granulosa cells with the application of the yeast two-hybrid screening. For ASB9 to interact with CKB in GC, we reasoned that the two proteins must be expressed at the same stage during follicular development. We have shown that CKB expression in GC is weak throughout the final stages of follicular development from small antral follicles to ovulatory follicles as compared to corpus luteum and at 6 hours post-hCG. In contrast, ASB9 was significantly induced by LH/hCG in preovulatory follicles with the strongest expression 24 hours post-hCG and was dramatically reduced in the CL. In rat ovary, although CKB activity was shown to be present throughout follicular development and luteinisation, it was found to be concentrated in the steroidogenic thecal and luteal cells of the ovary and to a much smaller extent in the granulosa [65]. Il is therefore conceivable that interaction between ASB9 and CKB proteins is minimized due to their different temporal expression pattern in relation to the physiological status of the granulosa cells.

## Conclusion

In this study, we demonstrated induction of ASB9 in granulosa cells of bovine ovulatory follicles and identified 10 potential binding partners for ASB9 including TNFAIP6 and HIF1A, which were further analyzed. However, there was no interaction between ASB9 and CKB in GC although CKB was shown as a target of ASB9 in other cell types. These data suggest a role of ASB9 in regulating the activity of target proteins likely by ubiquitination and proteasomal degradation. Using CRISPR-Cas9 approach, we showed that ASB9 inhibition leads to increased GC proliferation and may affect GC function by altering specific genes expression. Together, these results support a crucial role for ASB9 following hCG injection or the pituitary LH surge in the events leading up to ovulation and in the process of GC differentiation into luteal cells.

## Supporting information

S1 Fig Toxicity and autoactivation analyses of the pGBKT7-ASB9 construct. Y2HGold yeast strain were transformed with the empty vector (pGBKT7-EV) as control and the construct containing ASB9 (pGBKT7-ASB9). Cells were spread on SD/-Trp, SD/-Trp/X (X = X-alpha-Gal) and SD/-Trp/X/A (A = Aureobasidin A antibiotic) media for toxicity and autoactivation analyses. The construct ASB9 was not toxic to Y2HGold strain nor to Y187 strain. Also, ASB9 did not, by itself, activate the transcription of reporter genes (AUR-C, ADE2, HIS3, and MEL1), which would have resulted in blue colonies in the presence of Aureobasidin A.
(EPS)

S2 Fig Presence of zygotes as a result of Y2HGold[pGBKT7-ASB9] and Y187[pGADT7-Rec-OF cDNA] mating. An overnight culture of Y2HGold[pGBKT7-ASB9] was mated with 1 ml of the Y187[pGADT7-cDNA] library. The presence of zygotes (arrows) indicates a potential interaction between the bait (ASB9) and a prey contained in the library. These zygotes grow as blue colonies on a double dropout medium (medium lacking Leucine and Tryptophan) supplemented with X-alpha-Gal and aureobasidin antibiotic (DDO/X/A).
(EPS)

S3 Fig Representative result of blue colonies on DDO/X/A analyzed by yeast colony PCR. A slight portion of a blue colony from DDO/X/A was used to analyze for the presence or absence of an insert. The presence of an insert indicates a true positive suggesting a potential partner for ASB9 protein while the absence of an insert would have indicated a false positive. Blue colonies from DDO/X/A were further spread on the more stringent medium of quadruple dropout lacking Adenine, Histidine, Leucine, and Tryptophan and in the presence of the antibiotic aureobasidin (QDO/X/A). Plasmids were purified from true positive yeast colonies (grown on QDO/X/A), amplified by PCR and sequenced.
(EPS)

S1 File Data used for Figs 1A, 1B, 3, 4, 5A, 5B and 8. **(1A, 1B, 4, 5A, 5B, 8)** RT-qPCR experiments were performed using specific primers for each gene (listed in Table 1) and mRNA relative expression was calculated using the 2^-ΔΔCt^ method with *GAPDH* as reference gene. **(3)** Data generated using the ProLabel enzyme complementation assay. Luminescent signals (expressed in relative luminescent unit [RLU]) of TNFAIP6 and HIF1A were compared to a positive interaction (Pos. ctl), to an experimental control (Exp. ctl), and a negative control (Neg. ctl).
(DOCX)
