# Supplementary material for: Gonadotropin regulation of ankyrin-repeat and SOCS-box protein 9 (ASB9) in ovarian follicles and identification of binding partners
Source: PLoS One. 2019 Feb 27;14(2):e0212571. doi: 10.1371/journal.pone.0212571 (PMC6392328; doi:10.1371/journal.pone.0212571)
Supplement: S1 File — (1A, 1B, 4, 5A, 5B, 8) RT-qPCR experiments were performed using specific primers for each gene (listed in Table 1) and mRNA relative expression was calculated using the 2-ΔΔCt method with GAPDH as reference gene. (3) Data generated using the ProLabel enzyme complementation assay. Luminescent signals (expressed in relative luminescent unit [RLU]) of TNFAIP6 and HIF1A were compared to a positive interaction (Pos. ctl), to an experimental control (Exp. ctl), and a negative control (Neg. ctl). (DOCX) [file pone.0212571.s004.docx]

**Supplementary file**

1. Data used for figure 1A (*ASB9* relative mRNA expression and regulation).

| Normalized *ASB9* amount relative to OF 2^-ΔΔCt^ | | N |
| --- | --- | --- |
| SF | 1.82 +/-3.34 | 3 |
| DF | 0.96 +/-2.63 | 4 |
| OF | 100.00 +/-3.63 | 4 |
| CL | 1.46 +/-2.51 | 3 |

Abbreviations: SF, small follicles; DF, dominant follicles; OF, ovulatory follicles; CL, corpus luteum.

2. Data used for figure 1B (*ASB9* relative mRNA expression post-hCG injection).

| Normalized *ASB9* amount relative to 0 h 2^-ΔΔCt^ | | N |
| --- | --- | --- |
| 0 h | 10.00 +/-5.12 | 2 |
| 6 h | 15.73 +/-6.46 | 2 |
| 12 h | 56.94 +/-5.85 | 2 |
| 18 h | 62.44 +/-5.92 | 2 |
| 24 h | 102.13 +/-4.28 | 2 |

3. Data generated from chemiluminescence analyses and used for figure 3.

| Time (minutes) | HIF1A | TNFAIP6 | Neg. ctl | Pos. ctl | Exp. ctl |
| --- | --- | --- | --- | --- | --- |
| 0 | 0.000 | 0.000 | 0.000 | 0.000 | 0.000 |
| 5 | -9.670 | -3.967 | 2.033 | -12.000 | -0.967 |
| 10 | 3.933 | 15.833 | 4.033 | 94.000 | 4.033 |
| 15 | 15.833 | 21.733 | 5.433 | 98.670 | 1.033 |
| 20 | 40.500 | 42.000 | 5.000 | 101.330 | 2.000 |
| 25 | 85.567 | 104.767 | 2.467 | 125.000 | 1.067 |
| 30 | 87.000 | 152.300 | 12.900 | 242.330 | -0.400 |
| 35 | 158.167 | 180.467 | 26.267 | 360.670 | 3.967 |
| 40 | 220.967 | 296.567 | 8.967 | 472.670 | 11.867 |
| 45 | 257.500 | 345.000 | 18.800 | 710.000 | 9.900 |

Neg. ctl, Negative control; Pos. ctl, Positive control; Exp. Ctl, Experimental control (as described in the Material and Methods section)

4. Data used for figure 4 (*HIF1A* relative mRNA expression and regulation).

| Normalized *HIF1A* amount relative to OF 2^-ΔΔCt^ | | N |
| --- | --- | --- |
| SF | 36.10 +/-3.64 | 3 |
| DF | 103.55 +/-5.04 | 4 |
| OF | 100.00 +/-5.04 | 4 |
| CL | 27.79 +/-3.58 | 3 |

Abbreviations: SF, small follicles; DF, dominant follicles; OF, ovulatory follicles; CL, corpus luteum

5. Data used for figure 5A (*CKB* relative mRNA expression and regulation).

| Normalized *CKB* amount relative to OF 2^-ΔΔCt^ | | N |
| --- | --- | --- |
| SF | 17.91 +/-2.52 | 3 |
| DF | 9.154 +/-7.71 | 4 |
| OF | 10.00 +/-3.24 | 4 |
| CL | 143.74 +/-4.61 | 3 |

Abbreviations: SF, small follicles; DF, dominant follicles; OF, ovulatory follicles; CL, corpus luteum

6. Data used for figure 5B (*CKB* relative mRNA expression post-hCG).

| Normalized *CKB* amount relative to 0 h 2^-ΔΔCt^ | | N |
| --- | --- | --- |
| 0 h | 100.00 +/-7.17 | 2 |
| 6 h | 283.10 +/-9.79 | 2 |
| 12 h | 46.66 +/-8.82 | 2 |
| 18 h | 68.13 +/-9.79 | 2 |
| 24 h | 41.11 +/-7.85 | 2 |

7. Data used for figure 8 (*PCNA*, *CYP19A1*, *CYP11A1*, *CKB* mRNA expression).

|  |  | Normalized gene amounts relative to GC-Ctl 2^-ΔΔCt^ | N |
| --- | --- | --- | --- |
| *PCNA* | GC-Ctl | 103.88 +/-2.62 | 3 |
|  | GC-CRISPR | 115.48 +/-1.84 | 3 |
| *CYP19A1* | GC-Ctl | 16.99 +/-2.67 | 3 |
|  | GC-CRISPR | 20.38 +/-1.00 | 3 |
| *CYP11A1* | GC-Ctl | 100.00 +/-5.23 | 3 |
|  | GC-CRISPR | 128.82 +/-3.68 | 3 |
| *CKB* | GC-Ctl | 89.67 +/-5.55 | 3 |
|  | GC-CRISPR | 84.55 +/-6.37 | 3 |

Abbreviations: GC, granulosa cells; Ctl, control.
